# Questionable evidence for prospective effects of self-compassion and burnout on spiritual wellbeing: a simulated reanalysis and comment on Lee and Fung (2024)

**DOI:** 10.3389/fpsyg.2025.1576395

**Published:** 2025-04-30

**Authors:** Kimmo Sorjonen, Bo Melin

**Affiliations:** Karolinska Institutet (KI), Solna, Sweden

**Keywords:** burnout, cross-lagged panel model, self-compassion, simulation, spiritual wellbeing, spurious prospective effects, triangulation

## Abstract

**Objectives:**

The objective of the present simulated reanalysis was to scrutinize Lee and Fung's conclusion that self-compassion and burnout have causal effects on spiritual wellbeing.

**Methodology:**

We simulated data to resemble the data used by Lee and Fung. We used triangulation and fitted complementary models to the simulated data.

**Findings:**

We found contradictory increasing, decreasing, and null effects of initial self-compassion and burnout on subsequent change in spiritual wellbeing.

**Conclusion:**

The present divergent findings indicated that it is premature to assume causal effects of self-compassion and burnout on spiritual wellbeing and the suggestions by Lee and Fung in this regard can be challenged.

**Implications:**

It is important for researchers to be aware that correlations, including adjusted cross-lagged effects, do not prove causality in order not to overinterpret findings, something that appears to have happened to Lee and Fung. We recommend researchers to triangulate by fitting complementary models to their data in order to evaluate if observed effects may be due to true causal effects or if they appear to be spurious.

## Introduction

Burnout and wellbeing are highly relevant and tangible phenomena for many individuals. Parmar et al. (2022) found that emotional exhaustion, cynicism, and professional efficacy have a direct significant influence on job burnout. Ahmed et al. (2022), on the other hand, found a positive association between spirituality and psychological wellbeing that was mediated by job stress.

Lee and Fung (2024) analyzed longitudinal data (two waves of measurement, 8 months apart) on, for example, self-compassion, burnout, and spiritual wellbeing (i.e., perceived power and presence of God in everyday life) in a sample of clergy in Taiwan [*N* = 154, age range 26–72 years (*M* = 45 years), 56% women] with cross-lagged panel models. Lee and Fung reported a statistically significant positive cross-lagged effect of initial self-compassion, and a statistically significant negative cross-lagged effect of initial burnout, on subsequent spiritual wellbeing when adjusting for initial spiritual wellbeing. Lee and Fung interpreted these findings in causal terms, concluding, for example, that “the study highlights the causal impact of self-compassion on clergy's spiritual wellbeing” (p. 11).

However, it is well established that adjusted cross-lagged effects may be spurious due to correlations with residuals and regression to the mean (Campbell and Kenny, 1999; Castro-Schilo and Grimm, 2018; Sorjonen et al., 2019). For example, there appears to be a positive correlation between self-reported self-compassion and spiritual wellbeing (Lee and Fung, 2024), which could be due to a confounding impact by a third variable, e.g., general positivity. Therefore, we should expect that among individuals with the same initial spiritual wellbeing score, those with a higher self-compassion score have received a lower score on spiritual wellbeing compared with their true spiritual wellbeing, i.e., a more negative residual, while those with a lower self-compassion score have received a higher spiritual wellbeing score compared with their true score, i.e., a more positive residual. However, residuals tend to regress toward a mean value of zero between measurements. Consequently, we should expect a more positive, but spurious, change in spiritual wellbeing to a subsequent measurement among those with a higher initial self-compassion score compared with those with the same initial spiritual wellbeing score but with a lower initial self-compassion score.

For the sake of argument and in agreement with Lee and Fung, let us assume that a positive effect of initial self-compassion on subsequent spiritual wellbeing when adjusting for initial spiritual wellbeing can be interpreted as a causal increasing effect (not an interpretation we endorse). Then, for consistency, a positive effect of initial self-compassion on initial spiritual wellbeing when adjusting for subsequent spiritual wellbeing could be interpreted as a causal decreasing effect. This positive effect would suggest that high initial self-compassion had degraded high initial spiritual wellbeing to the same subsequent level as among individuals with lower initial spiritual wellbeing and lower initial self-compassion. Moreover, a positive, a negative, and a null effect of initial self-compassion on the subsequent—initial spiritual wellbeing difference score could be seen to suggest an increasing, a decreasing, and no causal effect, respectively. Similarly, a negative effect of initial burnout on subsequent spiritual wellbeing when adjusting for initial spiritual wellbeing, a negative effect of initial burnout on initial spiritual wellbeing when adjusting for subsequent spiritual wellbeing, and a null effect of initial burnout on the subsequent-initial spiritual wellbeing difference score, could be seen to suggest a decreasing, an increasing, an no causal effect of burnout on spiritual wellbeing, respectively.

The objective of the present study was to scrutinize the conclusion by Lee and Fung, based on findings in cross-lagged panel models, that self-compassion and burnout have causal effects on spiritual wellbeing. This objective was achieved by fitting complementary models to data simulated to resemble the data analyzed by Lee and Fung. To scrutinize claims and conclusions, and to reveal limitations of employed methods, are important parts of the scientific process. To the best of our knowledge, this is the first time the conclusion by Lee and Fung, that self-compassion and burnout have causal effects on spiritual wellbeing, has been scrutinized.

## Method

### Research design

The present study was a reanalysis of the study by Lee and Fung, using data simulated to resemble the data they analyzed, with the same sample size and correlations between variables. We used simulated data as the empirical data were not available to us. The corresponding author of the study by Lee and Fung did not respond to our request for the data. It should be noted that both the standardized effect of X on Y_2_ when adjusting for Y_1_ (Equation 1, Cohen et al., 2003) and on the Y_2_-Y_1_ difference (Equation 1, Guilford, 1965) are functions of correlations between the variables. Consequently, these effects will be the same in data, empirical or simulated, with the same correlations between variables and simulated data can be used to estimate what the effects would have been in corresponding empirical data. The original study by Lee and Fung employed an observational (i.e., non-experimental) two-wave longitudinal design.


(1) $$\begin{array}{l} E\left(\beta_{X,Y2.Y1}\right)=\frac{r_{X,Y2}-r_{X,Y1}r_{Y1,Y2}}{1-r_{X,Y1}^{2}} \end{array}$$

(2) $$\begin{array}{l} E\left(\beta_{X,Y2-Y1}\right)=\frac{r_{X,Y2}-r_{X,Y1}}{\sqrt{2\left(1-r_{Y1,Y2}\right)}} \end{array}$$

### Measures

In the present study, we used data simulated to resemble data on the measures used by Lee and Fung. In the original study, Lee and Fung measured (1) Self-compassion, with 6 items from the Self-Compassion Scale (SCS); (2) Burnout, with 9 items from the emotional exhaustion subscale of the Maslach Burnout Inventory (MBI); (3) Spiritual wellbeing, with the 6-item Clergy Spiritual Wellbeing Scale.

### Data collection method

We collected sample size and correlations between variables from the published paper by Lee and Fung (2024). The sample size (*N* = 154) was reported, for example, in the abstract on page 1. Correlations were reported in a table on page 6. In the original study, Lee and Fung collected data with an online survey on two occasions, eight months apart.

### Data analysis techniques

We used ordinary least squares (OLS) regression (Cohen et al., 2003) to estimate the following six standardized effects in data simulated to resemble the data used by Lee and Fung: (1) the effect of self-compassion at time 1 on spiritual wellbeing at time 2 when adjusting for spiritual wellbeing at time 1; (2) the effect of self-compassion at time 1 on spiritual wellbeing at time 1 when adjusting for spiritual wellbeing at time 2; (3) the effect of self-compassion at time 1 on the spiritual wellbeing at time 2—spiritual wellbeing at time 1 difference score; (4) the effect of burnout at time 1 on spiritual wellbeing at time 2 when adjusting for spiritual wellbeing at time 1; (5) the effect of burnout at time 1 on spiritual wellbeing at time 1 when adjusting for spiritual wellbeing at time 2; (6) the effect of burnout at time 1 on the spiritual wellbeing at time 2—spiritual wellbeing at time 1 difference score.

Analyses and the simulation for the present study were conducted with R 4.4.0 statistical software (R Core Team, 2025) using the MASS package (Venables and Ripley, 2002). The analytic script, which also generates the simulated data, is available at the Open Science Framework at https://osf.io/hr3tf/.

## Results

Initial self-compassion had a positive effect on subsequent spiritual wellbeing when adjusting for initial spiritual wellbeing [β = 0.183 (0.039; 0.327), *p* = 0.013], a positive effect on initial spiritual wellbeing when adjusting for subsequent spiritual wellbeing [β = 0.320 (0.190; 0.450), *p* < 0.001], and no statistically significant effect on the subsequent-initial spiritual wellbeing difference score [β = −0.085 (−0.245; 0.075), *p* = 0.295], respectively. These effects could be seen to suggest an increasing, a decreasing, and no causal effect of initial self-compassion on spiritual wellbeing, respectively. The second of these effects suggested that among individuals with the same subsequent degree of spiritual wellbeing (e.g., a standardized score of zero), those with high initial degree of self-compassion (e.g., 1) had had higher initial degree of spiritual wellbeing (1 × 0.320 = 0.320) compared with those with low initial degree of self-compassion (e.g., −1 and −1 × 0.320 = −0.320). Consequently, those with high initial degree of self-compassion had experienced a more negative change in spiritual wellbeing between the measurements (0–0.320 = −0.320) compared with those with the same subsequent degree of spiritual wellbeing but with lower initial degree of self-compassion [0–(−0.320) = 0.320]. This suggests that high initial self-compassion had degraded high initial spiritual wellbeing to the same subsequent level as among individuals with lower initial spiritual wellbeing and lower initial self-compassion.

Initial burnout had a negative effect on subsequent spiritual wellbeing when adjusting for initial spiritual wellbeing [β = −0.206 (−0.341; −0.072), *p* = 0.003], a negative effect on initial spiritual wellbeing when adjusting for subsequent spiritual wellbeing [β = −0.225 (−0.358; −0.092), *p* = 0.001], and no statistically significant effect on the subsequent-initial spiritual wellbeing difference score [β = 0.012 (−0.148; 0.172), *p* = 0.881], respectively. These effects could be seen to suggest a decreasing, an increasing, and no causal effect of initial burnout on spiritual wellbeing, respectively.

## Discussion

Burnout and wellbeing are highly relevant and tangible phenomena for many individuals. Parmar et al. (2022) found that emotional exhaustion, cynicism, and professional efficacy have a direct significant influence on job burnout. Ahmed et al. (2022), on the other hand, found a positive association between spirituality and psychological wellbeing that was mediated by job stress.

The present study set out to scrutinize the conclusion by Lee and Fung that self-compassion and burnout have causal effects on spiritual wellbeing. The present findings showed that the data analyzed by Lee and Fung could be used to support contradicting conclusions of increasing, decreasing, and null effects of initial self-compassion and burnout on subsequent change in spiritual wellbeing. Hence, the data cannot support any of those conclusions and the conclusions of causal effects by Lee and Fung appear premature and questionable.

### Theoretical and practical implications

It is important for researchers to bear in mind that correlations, including adjusted cross-lagged effects, do not prove causality in order not to overinterpret findings, something that appears to have happened to Lee and Fung. We recommend researchers to, as we did here, use triangulation by fitting complementary models to their data. If results from the models converge, conclusions of causality are corroborated (although never finally proven). If, on the other hand and as in the present case, findings diverge, conclusions of causality would appear premature.

### Limitations and potential areas of future studies

The data used by Lee and Fung, and simulated and reanalyzed here, were collected from a sample of clergy in Taiwan. It is unclear if the present findings, that it is premature to claim causal effects of self-compassion and burnout on spiritual wellbeing, apply to other cultural, social, and economic contexts.

As said above, the empirical data were not available to us and we used data simulated to resemble the data analyzed by Lee and Fung, with the same sample size and correlations between variables. Someone might perceive it as a major limitation that we used simulated data rather than the original empirical data. However, as said above, it is important to bear in mind that both adjusted regression effects and effects on difference scores are functions of correlations between variables (Equation 1 and Equation 2). This means that analyses of simulated data will identify the same effects as analyses of the empirical data with the same correlations between variables. Moreover, due to identical sample sizes, the statistical significance of the effects will also be the same in analyses of the simulated and the empirical data. As an analogy, if we know that *N*_1_ = 34, *M*_1_ = 27, *SD*_1_ = 9 in group 1 and *N*_2_ = 29, *M*_2_ = 22, *SD*_2_ = 8 in group 2 on some outcome of interest, respectively, we can estimate *T* = 2.31, *DF* = 61, *p* = 0.024, for the group difference, without access to individual-level data. It would be inconceivable to find a significantly lower score in group 1 compared with group 2 in analyses of the empirical individual-level data given these aggregated values, with a significantly higher score in group 1. Similarly, it would be inconceivable to find, for example, a negative effect of initial self-compassion on initial spiritual wellbeing when adjusting for subsequent spiritual wellbeing in analyses of the empirical individual-level data used by Lee and Fung, given the statistically significant positive effect found in the present analyses of simulated data with the same sample size and correlations between variables.

Future studies could scrutinize other concluded causal effects, based on cross-lagged panel models and reported in the research literature, in order to evaluate, as we did here, if the analyzed data could be used to support contradictory conclusions, which would suggest that conclusions of causality in the original study were premature.

## Conclusions

Lee and Fung (2024) concluded, based on analyses with cross-lagged panel models, that self-compassion and burnout have causal effects on spiritual wellbeing. However, in analyses of data simulated to resemble the data used by Lee and Fung, we found contradictory increasing, decreasing, and null effects of initial self-compassion and burnout on subsequent change in spiritual wellbeing. These divergent findings indicated that it is premature to assume causal effects of self-compassion and burnout on spiritual wellbeing and the suggestions by Lee and Fung in this regard can be challenged.

## Data Availability

Publicly available datasets were analyzed in this study. This data can be found here: The analytic script, which also generates the simulated data, is available at the Open Science Framework at https://osf.io/hr3tf/.
